# The rise of automated curiosity-driven discoveries in chemistry

**DOI:** 10.1039/d3sc03367h

**Published:** 2023-09-08

**Authors:** Latimah Bustillo, Teodoro Laino, Tiago Rodrigues

**Affiliations:** a Research Institute for Medicines (iMed), Faculdade de Farmácia, Universidade de Lisboa Lisbon Portugal tiago.rodrigues@ff.ulisboa.pt; b IBM Research Europe Säumerstrasse 4 8803 Rüschlikon Switzerland; c National Center for Competence in Research-Catalysis (NCCR-Catalysis) Zurich Switzerland

## Abstract

The quest for generating novel chemistry knowledge is critical in scientific advancement, and machine learning (ML) has emerged as an asset in this pursuit. Through interpolation among learned patterns, ML can tackle tasks that were previously deemed demanding to machines. This distinctive capacity of ML provides invaluable aid to bench chemists in their daily work. However, current ML tools are typically designed to prioritize experiments with the highest likelihood of success, *i.e.*, higher predictive confidence. In this perspective, we build on current trends that suggest a future in which ML could be just as beneficial in exploring uncharted search spaces through simulated curiosity. We discuss how low and ‘negative’ data can catalyse one-/few-shot learning, and how the broader use of curious ML and novelty detection algorithms can propel the next wave of chemical discoveries. We anticipate that ML for curiosity-driven research will help the community overcome potentially biased assumptions and uncover unexpected findings in the chemical sciences at an accelerated pace.

## Abstract 2

The quest for generating novel chemistry knowledge is critical in scientific advancement, and machine learning (ML) has emerged as an asset in this pursuit.

## Graphical abstract

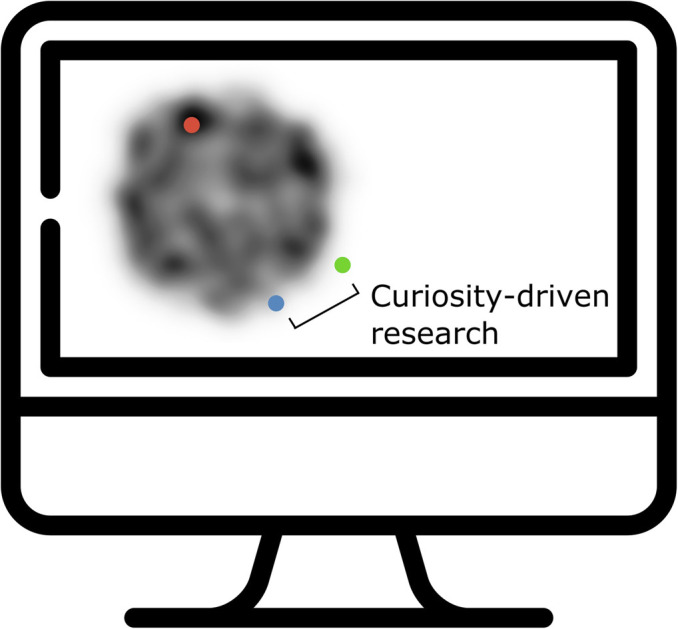

## Introduction

Chemistry, often referred to as the central science, plays a vital role in expanding knowledge and driving technological advancements across various domains. Discoveries in diverse subfields of chemistry are essential for developing life-saving drugs, diagnostics, and a wide range of materials that ultimately benefit society. Consequently, there is a constant demand for innovation and exploration of unconventional research paths that challenge established knowledge, which has led to a noticeable rise in the number of research manuscripts and patents.^1^ However, despite the exponential growth of publications over time, there has been a decline in their disruptive nature.^1^ Several factors may contribute to this trend. The lack of interdisciplinary collaboration,^2^ the dearth of the so-called ‘low-hanging fruit’, the ‘publish or perish’ culture that can determine the fate of scientific careers, and unstable funding policies have been argued^1^ as the main factors affecting creativity and discouraging the pursuit of unconventional and high-risk research avenues. In contrast, curiosity-driven basic research, *i.e.*, without pre-established or rigid goals, grants researchers the freedom to explore the unknown. A prime example of the power of curiosity-driven research is the serendipitous discovery of penicillin in 1928, which emerged from a curious observation and led to the antibiotic therapy revolution.^3,4^

**Fig. 1:**
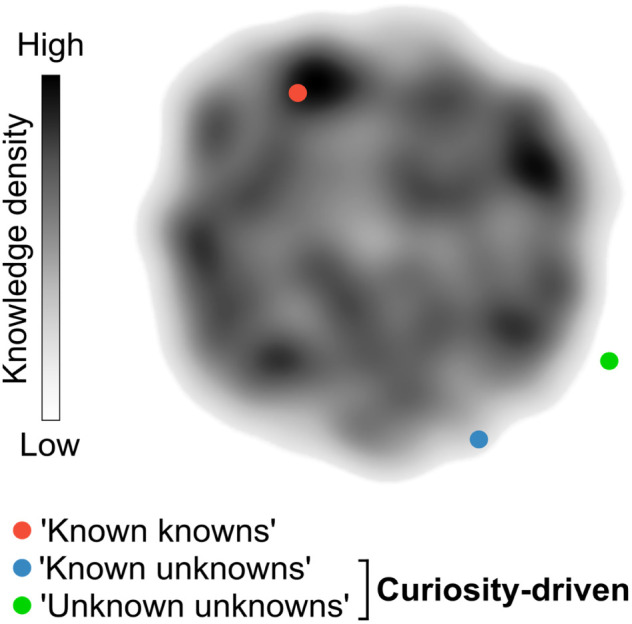
Curiosity-driven research as a strategy to pursue the ‘unknown’. Research conducted in areas that are densely populated by prior work (orange) serves to consolidate intuition and contributes to incremental gains in knowledge. In contrast, research conducted in sparsely populated areas of the search space (green and blue) pushes the boundaries of knowledge. These endeavours are more likely to yield disruptive outcomes, reshaping the state-of-the art and even giving rise to entirely new fields of research.

Curiosity promotes the discovery of remarkable and unconventional findings that can lay the foundation for future advancements, sometimes decades or even centuries after the original results. While curiosity is a subjective concept, it can be loosely defined as the ability to pose and pursue research questions when outcomes are unpredictable or uncertain. Curiosity's primary aim is to unravel the ‘unknown unknowns’ of science. However, curiosity can also lead to unexpected discoveries when researching well-defined questions with the goal of consolidating knowledge (‘known knowns’) or exploring contiguous, yet uncharted search spaces (‘known unknowns’; Fig. 1). Importantly, curiosity-driven research in basic or foundational discovery programs may not intend to solve problems immediately. Instead, such research programs can generate knowledge and often provide tools that disrupt conventional thinking and inspire future experimental approaches. This also means that the rewards for curiosity-driven research may have diverse and sometimes prolonged timelines, which can be discouraging for researchers advancing their careers.^1^ Conversely, research aimed at closing knowledge gaps, consolidating information, or exploiting existing knowledge is less likely to challenge established intuition, although being equally valuable. Striking the right balance between both modalities is crucial to ensure the sustainability of research programs.

In the last years, the generation of novel research hypotheses is strongly benefitting from automated data analysis and pattern recognition. Here, machine learning (ML) algorithms enable scientists to accelerate their understanding of complex phenomena. The resurgence of ML in chemistry has been fuelled by increased storage capacity, powerful hardware, and a wealth of data to delve on. Tasks that were once demanding to machines, *e.g.*, retrosynthetic planning,^5–7^ molecular design,^8–10^ prediction of protein structure and function,^11–13^ prediction of biological activity^14–16^ and others,^17–19^ have now become significantly more attainable and generalizable, providing valuable assistance to bench chemists.^20^ ML workflows and pipelines are primarily implemented to accelerate chemical research, with the scope of prioritizing experiments with high confidence and likelihood of success, while minimizing exploration towards pre-established objectives. This exploitative approach, although efficient, is inherently cautious and risk averse. Thus, the current use of ML approaches for experiment selection may confine research within a limited search space, although connecting ‘known knowns’ in new ways. We speculate that the potential applications of ML tools extend beyond their current scope. Not only can ML be utilised to augment knowledge through ‘artificial curiosity’ and ‘creativity’, but it can also serve as a strategy to explore vast search spaces in an open-ended manner.^21^

With this perspective, we wish to promote a stimulating conversation on the use of ML to support curiosity-driven research, firmly rooted in probabilistic principles. We illustrate our viewpoints by highlighting specific use cases from the existing literature. Furthermore, we delve into the significance of low data scenarios and uncertainty as catalysts for curiosity and emphasize the pressing need to embrace, document and rationalize ‘negative’ results in chemistry that stem from risk-taking research. We advocate for the adoption and prospective evaluation of one-/few-shot learning, novelty/anomaly detection, and reinforcement or active learning methodologies as versatile approaches to inform curiosity-driven research. Finally, we anticipate that these methodologies can help unveil previously ‘unknowns’ in discovery chemistry, overcome biased human intuition, and expedite unexpected findings in the era of digital chemistry.

### Randomness, uncertainty and chemical discovery

**Fig. 2:**
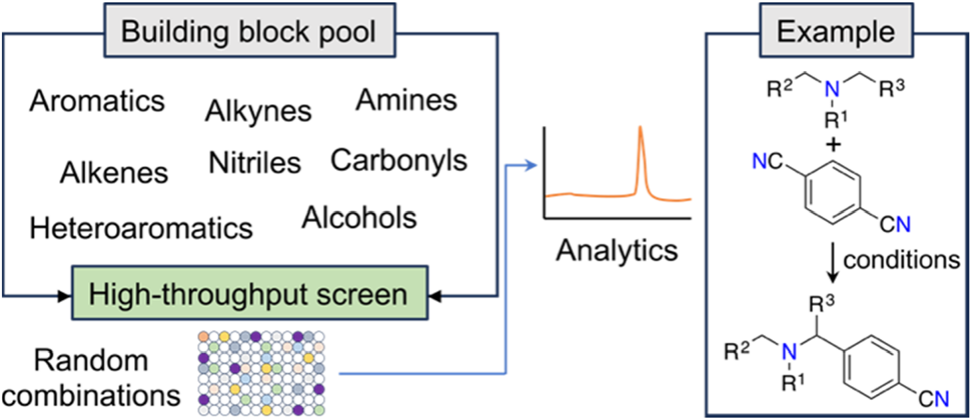
Workflow for the discovery of a photoredox C–H arylation reaction. The random selection of reaction partners led to the serendipitous discovery of a novel reaction.

New and unconventional findings in synthetic chemistry provide opportunities to access previously elusive molecules. Large scale, randomized assays have been proposed as a strategy to expedite the occurrence of serendipitous discoveries. This approach assumes that brute force experimentation using specialized equipment enhances the probability of uncovering novel discoveries in non-linear search spaces (*e.g.*, ref. 22). A notable example of this open-ended approach is the fortuitous identification of an innovative α-amino C–H arylation transformation^22^ by MacMillan and co-workers, which holds significant potential for advancing drug development (Fig. 2). Serendipity — making unexpected and surprising discoveries — is taxonomically complex.^23^ While it can be targeted, it ultimately emerges from curiosity and the inability to anticipate research outcomes.^23^ From a ML point of view, curiosity and serendipitous discoveries might be linked to high predictive uncertainty. Harnessing the power of serendipity has been historically challenging to chemists, although recent advancements have shown an intriguing reversal of this trend.^24,25^

In a recent study, Schrier^26^ and colleagues proposed a novel approach combining ML with a serendipity-based recommendation system. Using active learning strategies, *i.e.*, iterative experiment selection, the ML tool aimed to strike a balance between prediction accuracy (exploitation) and curiosity (exploration). The approach utilised distance metrics within the search space to assess the diversity and novelty of the proposed experiments, thereby enhancing exploration. This mathematical formalization of ‘curiosity’ enabled the sampling and testing of chemical compositions containing three different solutes (metal halide, amine and formic acid) with the aid of a robotic liquid handler. Within a discretised search space consisting of approximately 20 000 feasible experiments, some of the performed assays resulted in the formation of crystals, which was the targeted endpoint in the study. As suggested by the authors, the implemented curiosity-driven method is ideally suited to overcome historical data distributions in training sets, which often incorporate anthropogenic and selection biases.^27^ Indeed, the influence of data distributions and their evolution over the course of a project timeline are crucial yet often overlooked factors in the deployability of ML models.^28^ When combined with selection biases, these factors have the potential to undermine the accuracy of ML pipelines.^27^ Additionally, they pose significant challenges in terms of managing expectations regarding model performance. We expect that such limitations can be partially mitigated through the adoption of a ‘curious’ ML approach that focuses on improving model performance through dynamic updates.

**Fig. 3:**
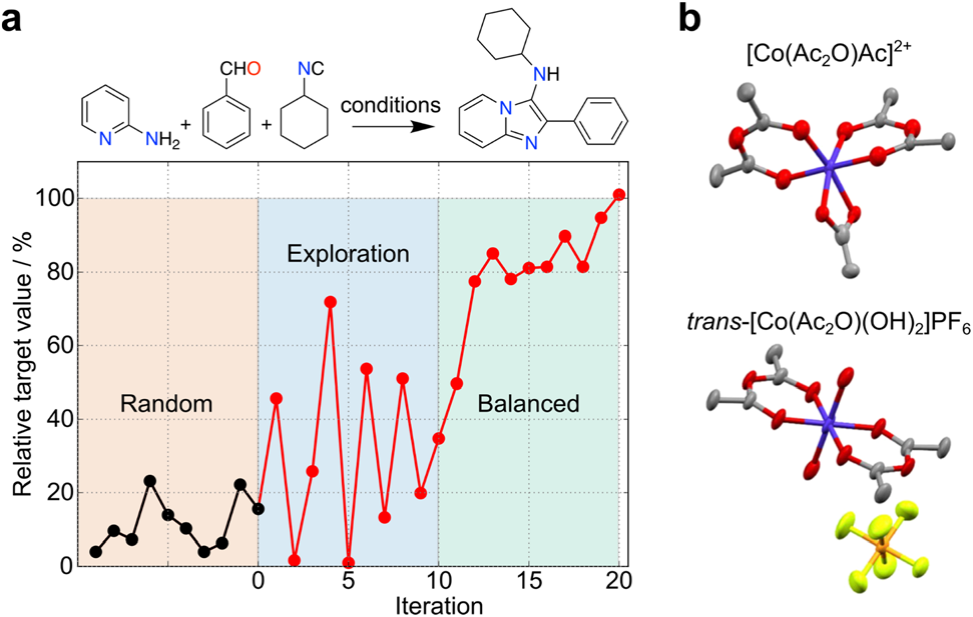
Exploration of search spaces with curiosity-driven approaches. (a) Optimization of conditions for a Ugi reaction with curiosity-driven machine learning (ML).^29^ Starting from an initial set of 10 random reactions, the ML algorithm iteratively suggest reactions with the highest uncertainty, irrespective of the predicted yield (iterations 1–10). The iterative selection is carried out with the goal of improving model performance by sampling different regions of the search space. The ML tool then adopts a balanced approach (iterations 11–20), selecting reactions with the highest uncertainty, among those with the highest predicted yield. (b) An unconstrained exploration of the search space based on mass spectrometry data (expected *vs.* experimental) was implemented to quantify serendipity. Using this method, cobalt(iii) anhydride complexes were discovered.^30^ Co, dark blue; C, gray; O, red; N, light blue; P, orange; F, light green.

The uncertainty in experimental results can propagate over time, but also open unforeseen research lines and opportunities for pushing the boundaries of knowledge. These outcomes contain valuable and informative content, which can reshape decision-making processes in ML and foster innovation. In another example, simulated curiosity was embedded into random forests to optimize the conditions of a tool 3-component Ugi reaction^29^ (Fig. 3a). Despite uncertainties in yield measurements and resulting prediction errors, a small set of pseudo-random experiments served as an ideal starting point for exploring reactivity spaces. A constraint-free approach driven by ‘curiosity’ resulted in a seemingly unpredictable behaviour in respect to reaction selection and outcomes. As the ML model gained a better understanding of the reactivity, the behaviour gradually stabilized, leading to a balanced exploration/exploitation strategy. Also, Bayesian optimization with a Gaussian process surrogate model was employed to discover novel coordination chemistry. Here, the metric relied on comparing the experimental and starting material's spectra, wherein larger differences indicated a higher likelihood of identifying a novel outcome^30^ (Fig. 3b). Along these lines, we anticipate that serendipity, as a measure of novelty, can be formalized through different metrics and incorporated into multiple ML algorithms with the goal of boosting research efficiency and as a viable alternative to brute force, random experimentation. The choice of algorithm must however be adapted to the chemistry problem in hand, namely the amount and type of data (*e.g.*, sparsity).

**Fig. 4:**
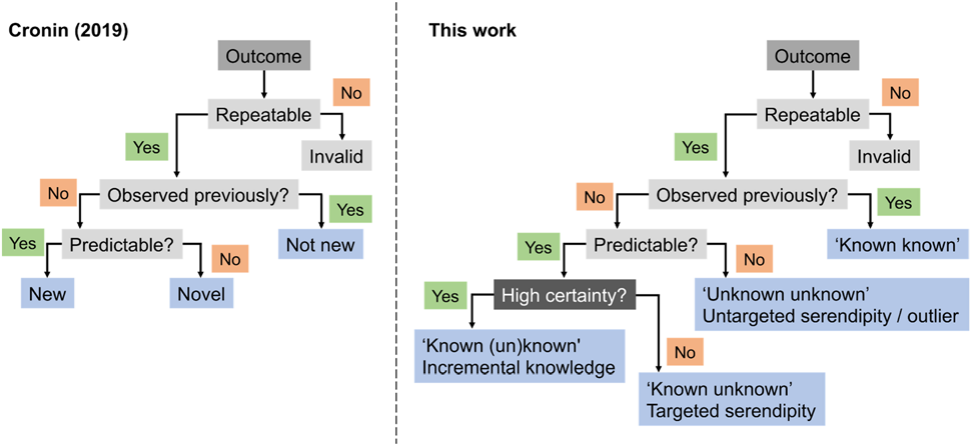
Flow chart for the categorization of discoveries in respect to their curiosity-driven approach. A corollary for curiosity-driven research is the underlying uncertainty in the experimental output and the associated gains for expert intuition. Surprising findings can be actively sought after (‘known unknowns’) or fortuitously identified (‘unknown unknowns’).

In 2019, Cronin and co-workers proposed heuristics for a definition of ‘newness’ and ‘novelty’.^31^ According to their framework, an experimental output would be categorized as ‘new’ if it had not been previously observed but could be predicted. If it had not been observed and was equally unpredictable, then it would be ‘novel’, akin to an outlier. Building on this concept, we here expand the ontology by introducing serendipity, ML predictive uncertainty and contribution to expert intuition (Fig. 4), as discussed above, and as distinguishing factors for categorizing discoveries and research approaches. While it is apparent that the highest gains arise from high-risk and curiosity-driven research, the main question now lies in identifying viable starting points to navigate the unknown.

### Low data, ‘failed’ experiments and information as curiosity catalysts

Surprising discoveries inherently occur in unexplored spaces where realistic predictions of experimental output are hindered by the lack of available data. A key challenge for current ML toolkits is to develop robust predictive models with limited (low) data while effectively addressing observational and model uncertainties.^32^ Working with real-world data, *i.e.*, either scarce, sparse,^33^ unbalanced^34^ and/or incomplete^35^ remains the ultimate challenge and the most common scenario in ML for chemistry. We envision that low data scenarios offer opportunities for targeted and untargeted serendipity (Fig. 4) and thus serve as an ideal starting point for curiosity-driven ML research. By simulating curiosity, ML can promote a stepwise exploration of search spaces, while pushing knowledge boundaries and improving its domain of applicability. The focus is on generating knowledge by prioritizing the next most informative experiments. We argue that information indeed plays a vital role in this context. It has been established that having more data does not always correlate with better model performance, but selecting the most informative experiments usually does.^36^ By formalizing simulated curiosity, *e.g.*, according to information theory principles, one is more likely to meet surprising findings and unravel the ‘unknowns’ in the chemical sciences. Some of these concepts have been recently highlighted by Reker and colleagues, who demonstrated that ML models trained with less than 50% of the total available training data can outperform their counterparts trained on the full dataset for tasks, such as bioactivity (*e.g.*, BACE or HIV) and physicochemical property (*e.g.*, cross blood–brain barrier) prediction.^36^ This achievement is made possible by curated knowledge bases that minimize biases and redundant information. Keeping unique molecules for model development was cornerstone towards that end and was attained through molecular substructure diversity.

**Fig. 5:**
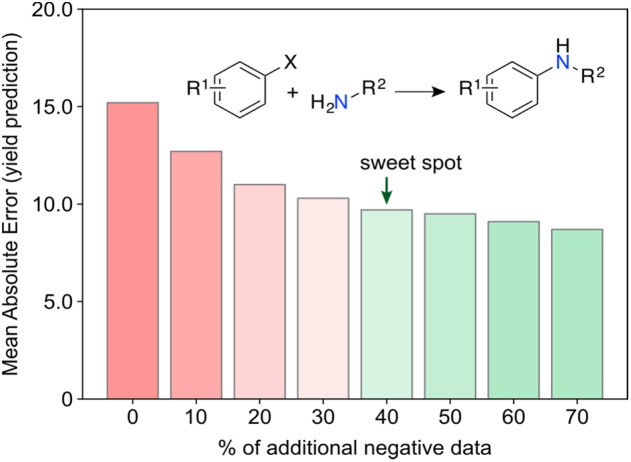
Machine learning (ML) models for the prediction of Buchwald–Hartwig cross-coupling yields. ML models using biased datasets (positive data, *i.e.*, high yielding reactions) underperform relative to counterparts that include a larger amount of ‘negative’ (low yielding) reactions. Evidence suggests that ‘negative’ data holds significant information up to a certain point (∼40% additional data), beyond which the improvements in ML performance become less significant. This finding emphasizes the critical need of reporting ‘failed’ experiments in chemistry. Predictive ML tools with balanced datasets can then be more effectively employed to explore search spaces through curiosity-driven approaches.

Aside from information, we argue that ‘negative’ data is another import catalyst for curiosity. High-risk and exploratory research programs often yield a substantial number of ‘negative’ results from experiments deemed unsuccessful, *i.e.*, results that are either undesired or contrary to expectations. However, from a philosophical perspective, one can argue that no experiment truly fails or wastes resources if information is extracted, or new insights are gained. This underlying message is conveyed in a recent work by Janela and Bajorath,^37^ where multiple ML models for bioactivity prediction of small molecules failed to demonstrate improved performance when compared to a simple nearest neighbour analysis. Albeit underwhelming, the result is highly relevant. Together with other cases,^38,39^ it pinpoints the importance of understanding dataset limitations and conducting control experiments^28^ to realistically assess the expected baseline performance of ML tools. In an independent study, Grisoni and co-workers reached similar conclusions by revealing significant limitations in state-of-the-art representation learning approaches when trained under low data regimes.^40^ Altogether, we argue the expression ‘negative data’ is an oxymoron when the results positively impact decision-making processes and enhance chemical intuition. For example, it has been shown that regression ML models can improve their performance with the addition of artificially generated ‘negative’ data^41^ (Fig. 5). Using the Buchwald–Hartwig reaction and Suzuki–Miyaura coupling as use cases, Glorius and co-workers alerted for an existing reporting bias towards high-performing reactions indexed in Reaxys. Despite a range of ML models and molecular representations yielding subpar accuracy on this biased data, the introduction of small amounts of ‘negative’ data (20–40%) led to significant improvements in model performance. This substantiates the notion that ‘negative’ examples can be highly informative^42^ and, at times, hold more significance for model inclusion and performance than redundant ‘positive’ data. It is thus conceivable that ‘negative’ results can act as catalysts for unexpected discoveries by enhancing ML models. Ultimately, not reporting ‘negative’ results further contributes to the sparse coverage of search spaces. That skewed coverage of input space and all possible outputs (due to selection and reporting bias) poses risks that can stall current enthusiasm for ML. Yet, the adoption of a curiosity-driven ML approach offers a promising solution to mitigate these shortcomings and foster the generation of new chemical intuition.

### Machine learning strategies for curiosity-driven research

We argue that the advancement of curiosity-driven research in chemistry requires a new ML toolkit, with a particular emphasis on unsupervised and self-supervised learning methods. Alternatively, state-of-the-art ML algorithms may be used with customised decision functions and experiment selection policies that account for uncertainty. Active and reinforcement learning have already demonstrated their value in scenarios where available data is insufficient to build robust probabilistic models. Different ML techniques, including random forests, support vector machines, deep neural networks and Bayesian optimization hinging on Gaussian processes as surrogate model have found widespread application in chemistry. With the exception of neural networks, the popularity of the remaining methods likely lies in their ease of implementation, tuning and the ability to work with low data. Again, the key is adopting an exploratory approach that selects experiments contributing most effectively to enhancing model performance. Random forests, support vector machines and Bayesian optimization provide easy access to predictive uncertainty estimates, which are critical to leverage a curiosity-driven research approach. In random forests – a decision tree ensemble method – this is typically accomplished by assessing predictive variance for regression models or class probabilities for classifiers. In support vector machines – a method that identifies a separating plane in N-dimensional space – the lower distance of the test data to the hyperplane indicates higher predictive uncertainty, and in Bayesian optimization a 95% confidence interval can be calculated. Finally, in reinforcement learning with recurrent neural networks, sequential actions aim at maximizing a reward function tailored to specific project requirements. While we speculate all these supervised learning approaches are well-established, in this section we wish to highlight underutilized methods in the chemical sciences that hypothetically possess equal or greater suitability for low data and curiosity-driven research.

**Fig. 6:**
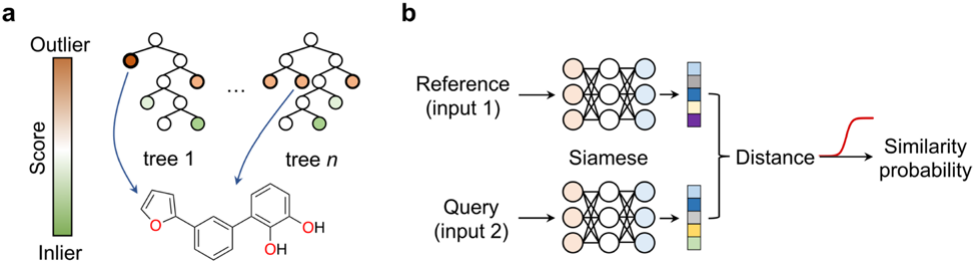
Machine learning approaches for low data and curiosity-driven research. (a) Isolation forests are used to identify anomalies/outliers within a descriptor space. Anomalies (*e.g.*, molecule) present shorter decision paths compared to inliers. (b) Siamese neural networks offer a solution for one-/few-shot learning. They consist of identical networks that encode both a reference and a query. The distance between the embeddings/representations correlates with the similarity probability, which is obtained through an activation function (*e.g.*, sigmoid; red).

Anomaly, novelty or outlier detection methods are different designations for a rather large group of disparate unsupervised or self-supervised ML algorithms that make predictions solely based on the structure of the descriptors. The goal of anomaly detection methods is identifying events deviating from the norm,^43^*i.e.*, from a bell-shaped distribution. While anomaly detection methods have been extensively explored in areas like image classification and fraud detection, their application in the chemical sciences has been relatively limited. Two common and established use cases include the identification of faulty instruments^44^ in chemical plants and sensor monitorization^45–47^ in process control. In these cases, the methods are used on time-series data, where the event of interest is often underrepresented or absent in the training data. Nonetheless, anomaly detection methods can be applied to various types of data formats, including tabular data, and present diverse architectures, *e.g.*, tree-based methods or neural networks. This class of ML methods may be ideally suited to push the boundaries of knowledge and uncover ‘unknown unknowns’ in the chemical sciences. A recent study reported a deep learning-based anomaly detection method to identify molecules with different graph characteristics within publicly available datasets.^48^ Graph neural networks use node (atom) and edge (bond) features to dissect chemical structures. When combined with contrastive learning they were employed to distinguish normal from abnormal graphs, while effectively addressing class imbalances and the scoring task. Specifically, said anomaly detection method implemented a dual-graph encoder, which involved encoding and decoding the real molecular graph with a graph convolutional autoencoder to learn node representations. Additionally, nodes were perturbed with Gaussian noise to learn an alternative graph representation. Contrastive learning was then applied to enhance the different representations and scored using contrastive learning loss. We speculate that similar methods based on variational autoencoders^49^ that sample and reconstruct from a probability density or the so-called latent space, or siamese neural networks^50^ (see below) can be employed and may hold promise in different aspects of medicinal chemistry: (1) they may flag under-represented chemotypes that are worth exploring further, thus promoting the development of innovative chemical matter and (2) they may flag unusual molecules and identify outliers in screening libraries, thus suggesting their re-testing. These concepts may actually have a broader applicability across the chemical sciences, including the promotion of reaction discovery, catalyst, drug and protein design among others. Alternatively, isolation forests can also be employed and may provide interesting baseline models for anomaly detection. In essence, this unsupervised method operates similarly to random forests; the average number of splits needed to isolate an example indicate their dissimilarity to the remaining examples. Anomalies are easier to isolate and require fewer splits (Fig. 6a). This concept can be applied to identify low solubility small molecules, which tend to aggregate in aqueous solutions and frequently lead to false positive readouts in primary biological screens.^34^ Rather surprisingly, the formation of said undesirable aggregates is insufficiently controlled for and reported in the literature, making them a form of ‘negative’ data. Still, their early detection can mitigate attrition^51^ in drug discovery pipelines. With an optimized isolation forest model, it was possible to flag 52% of the experimentally confirmed aggregating small molecules (the minority class/anomaly) and 79% of non-aggregating entities in a test set.^52^ While the result may not be perfect, it suggests that anomaly detection algorithms can serve as valuable decision-making tools in low data regimes and support curiosity-driven research.

Most of the currently celebrated deep learning algorithms are data hungry, highlighting a mismatch between the low data availability in chemistry and the capabilities^53^ of available technologies. Siamese neural networks offer a solution to address data scarcity and enhance knowledge in areas where training examples are rare. Siamese neural networks can employ different architectures, *e.g.*, convolutional, long–short term memory cells^53^ or the more recent transformers.^54^ Regardless of the architecture, siamese networks learn a distance metric in the input space to classify two events as (non-)identical, in a process known as one-/few-shot learning depending on the amount of training data available.^55^ In essence, the examples' descriptors are processed through two (siamese) neural networks that were trained on the same data and thus share interlayer weights.^50^ Objects with related characteristics will have similar representations, resulting in lower distance values. This concept has gained interest in the chemistry community as it enables the exploration of uncharted research questions and delving into descriptor structures without relying solely on abundant target labels. In practice, such an approach alleviates the need for extensive and expensive experimentation. Prominent examples include the prediction of bioactivities,^55,56^ relative binding energies,^57^ physicochemical properties with uncertainty estimation,^58^ among others. However, it is important to note that the utility of these emerging methods in chemistry has mainly been demonstrated retrospectively or in pseudo-prospective scenarios with simulated real-world use cases. We argue that, albeit promising, further assessment will require its application to real world research programs, embracing the possibility of encountering unexpected results. Only then one can truly gauge which methods are more promising to support curiosity-driven research.

## Outlook

In this perspective, we explored the philosophy of using ML to support curiosity-driven research. This approach contrasts the mainstream use of ML models that prioritize experiments according to exploitative goals and are focused on ‘positive’ outcomes. We also emphasized its potential for tapping ‘serendipity’ – in opposition to random experimentation – and accessing innovative discoveries in chemistry. To fully harness the potential of ML and uncover the ‘unknowns’ of chemistry, we anticipate that the community will start placing a stronger emphasis into small and higher quality datasets that include ‘negative’ experimental outcomes. The well-known low data roadblock hinders the use of ML for exploitation, but it can be reframed as an opportunity for knowledge augmentation. While more data does not necessarily guarantee better performing models, higher quality and informative results often do. Further, discoveries in unexplored areas are more prone for disruption and value creation, which may come at a cost of higher failure rates. With that in mind, one can anticipate adjustments to the current ML toolkit to accommodate new research approaches that simulate human ‘curiosity’. Namely, the design of tailored decision functions and the implementation of bespoke unsupervised and/or self-supervised learning algorithms are inevitable to deal with sparsely populated search spaces more efficiently. The proposed mentality shift is already ongoing and expected to accelerate through the recent constitution of international consortia. The applications of anomaly/novelty detection and one-/few-shot contrastive learning are only the first of many examples to come. We consider these approaches as defining steppingstones for the exploration of the ‘unknown’. We also believe that ML supporting curiosity-driven research has the potential to disrupt existing knowledge boundaries and assist expert intuition formulating new research questions through unexpected observations. Given the generalized interest in understanding machine made decisions, it will be important to explore the roots of ML decision-making processes and simulated curiosity. With a healthy dose of scepticism, we envision that by combining the discussed approaches with elements of causal learning, *e.g*., counterfactuals, we can usher in a new era of digital chemistry, foster unexpected discoveries and trust, and mitigate biases. These insights will be instrumental in bridging the gap between simulated curiosity and human intuition, an aspect that will be essential in driving future generations of theoretical and wet laboratory chemists.

## Author contributions

All authors contributed to writing this perspective.

## Conflicts of interest

T. Rodrigues is co-founder and shareholder of TargTex S.A., and a consultant to the pharmaceutical/biotechnology industry.

## Supplementary Material
